# Severe NAD(P)HX Dehydratase (NAXD) Neurometabolic Syndrome May Present in Adulthood after Mild Head Trauma

**DOI:** 10.3390/ijms24043582

**Published:** 2023-02-10

**Authors:** Nicole J. Van Bergen, Karen Gunanayagam, Adam M. Bournazos, Adhish S. Walvekar, Marc O. Warmoes, Liana N. Semcesen, Sebastian Lunke, Shobhana Bommireddipalli, Tim Sikora, Myrto Patraskaki, Dean L. Jones, Denisse Garza, Dale Sebire, Samuel Gooley, Catriona A. McLean, Parm Naidoo, Mugil Rajasekaran, David A. Stroud, Carole L. Linster, Mathew Wallis, Sandra T. Cooper, John Christodoulou

**Affiliations:** 1 Brain and Mitochondrial Research Group, Murdoch Children’s Research Institute, Royal Children’s Hospital, Melbourne, VIC 3002, Australia; 2 Department of Paediatrics, University of Melbourne, Melbourne, VIC 3002, Australia; 3 Department of Neurology, Royal Hobart Hospital, Hobart, TAS 7000, Australia; 4 Kids Neuroscience Centre, The Children’s Hospital at Westmead, Westmead, NSW 2145, Australia; 5 The Children’s Medical Research Institute, 214 Hawkesbury Road, Westmead, Sydney, NSW 2145, Australia; 6 Enzymology and Metabolism Group, Luxembourg Centre for Systems Biomedicine, University of Luxembourg, L-4367 Belvaux, Luxembourg; 7 Department of Biochemistry & Pharmacology, Bio21 Molecular Science & Biotechnology Institute, University of Melbourne, Melbourne, VIC 3002, Australia; 8 Victorian Clinical Genetics Services, Royal Children’s Hospital, Melbourne, VIC 3002, Australia; 9 School of Medicine, University of Tasmania, Hobart, TAS 7000, Australia; 10 Tasmanian Clinical Genetics Service, Royal Hobart Hospital, Hobart, TAS 7000, Australia; 11 Department of Anatomical Pathology, Alfred Hospital, Melbourne, VIC 3002, Australia; 12 Department of Medical Imaging, Royal Hobart Hospital, Hobart, TAS 7000, Australia; 13 Discipline of Child and Adolescent Health, Faculty of Health and Medicine, University of Sydney, Sydney, NSW 2006, Australia

**Keywords:** NAXD deficiency, NAXE deficiency, metabolite repair, metabolism, febrile illness, oral ulcers, skin lesions, neurodegeneration, myoclonic encephalopathy

## Abstract

We have previously reported that pathogenic variants in a key metabolite repair enzyme NAXD cause a lethal neurodegenerative condition triggered by episodes of fever in young children. However, the clinical and genetic spectrum of NAXD deficiency is broadening as our understanding of the disease expands and as more cases are identified. Here, we report the oldest known individual succumbing to NAXD-related neurometabolic crisis, at 32 years of age. The clinical deterioration and demise of this individual were likely triggered by mild head trauma. This patient had a novel homozygous *NAXD* variant [NM_001242882.1:c.441+3A>G:p.?] that induces the mis-splicing of the majority of *NAXD* transcripts, leaving only trace levels of canonically spliced *NAXD* mRNA, and protein levels below the detection threshold by proteomic analysis. Accumulation of damaged NADH, the substrate of NAXD, could be detected in the fibroblasts of the patient. In agreement with prior anecdotal reports in paediatric patients, niacin-based treatment also partly alleviated some clinical symptoms in this adult patient. The present study extends our understanding of NAXD deficiency by uncovering shared mitochondrial proteomic signatures between the adult and our previously reported paediatric NAXD cases, with reduced levels of respiratory complexes I and IV as well as the mitoribosome, and the upregulation of mitochondrial apoptotic pathways. Importantly, we highlight that head trauma in adults, in addition to paediatric fever or illness, may precipitate neurometabolic crises associated with pathogenic *NAXD* variants.

## 1. Introduction

NADH and NADPH are cofactors that are central to many metabolic processes, including glycolysis, the Krebs cycle, fatty acid β-oxidation, the mitochondrial electron transport chain, the pentose phosphate pathway and fatty acid synthesis [1]. When hydrated, the damaged R- and S-NAD(P)HX metabolites are redox inactive and interfere with these processes. Two highly conserved enzymes, NAD(P)HX dehydratase (NAXD) and NAD(P)HX epimerase (NAXE), repair these damaged metabolites. Pathogenic variants in the *NAXD* [2] and *NAXE* [3] genes are associated with rapid clinical demise in young children after encountering an otherwise trivial stress (fever, infection or illness). Early reports of NAXD deficiency were constrained to paediatric cases [2] but the clinical and genetic spectrum of NAXD deficiency is broadening [1]. Herein, we report the oldest known individual succumbing to NAXD-related neurometabolic crisis, whose clinical deterioration and demise were likely triggered following head trauma.

## 2. Case Presentation

### 2.1. Proband

A 32-year-old male of Indian origin, born to non-consanguineous parents, initially presented to hospital with post-concussive symptoms of headaches and episodic vertigo. One week prior, he had a low-speed motor vehicle accident with head strike but no loss of consciousness. In the months leading up to this, he had developed mild headaches, unsteadiness, subtle behavioural changes and pruritis.

He had a history of poorly controlled childhood focal epilepsy, including one prolonged admission at the age of four, with oral ulcerations, ‘encephalitis’ and right-hand paresis. In adolescence and early adulthood, he had improved control on carbamazepine and phenytoin, which were ceased prior to migrating to Australia in his mid-20s. However, he had ongoing episodic oral mucositis, managed with intermittent vitamin supplements (B12, folate and nicotinamide) until cessation 6 months prior to presentation.

In the following weeks, he developed rapidly worsening cognitive and behavioural disturbance, multidirectional nystagmus, ataxia, progressive myoclonic twitches affecting the face and upper limbs as well as autonomic dysfunction with tachycardia, urinary retention and bowel ileus. He had skin biopsy-proven superficial perivascular dermatitis. Weeks later, he developed severe oral mucositis and was intubated due to a threatened airway and progressive encephalopathy.

**Figure 1:**
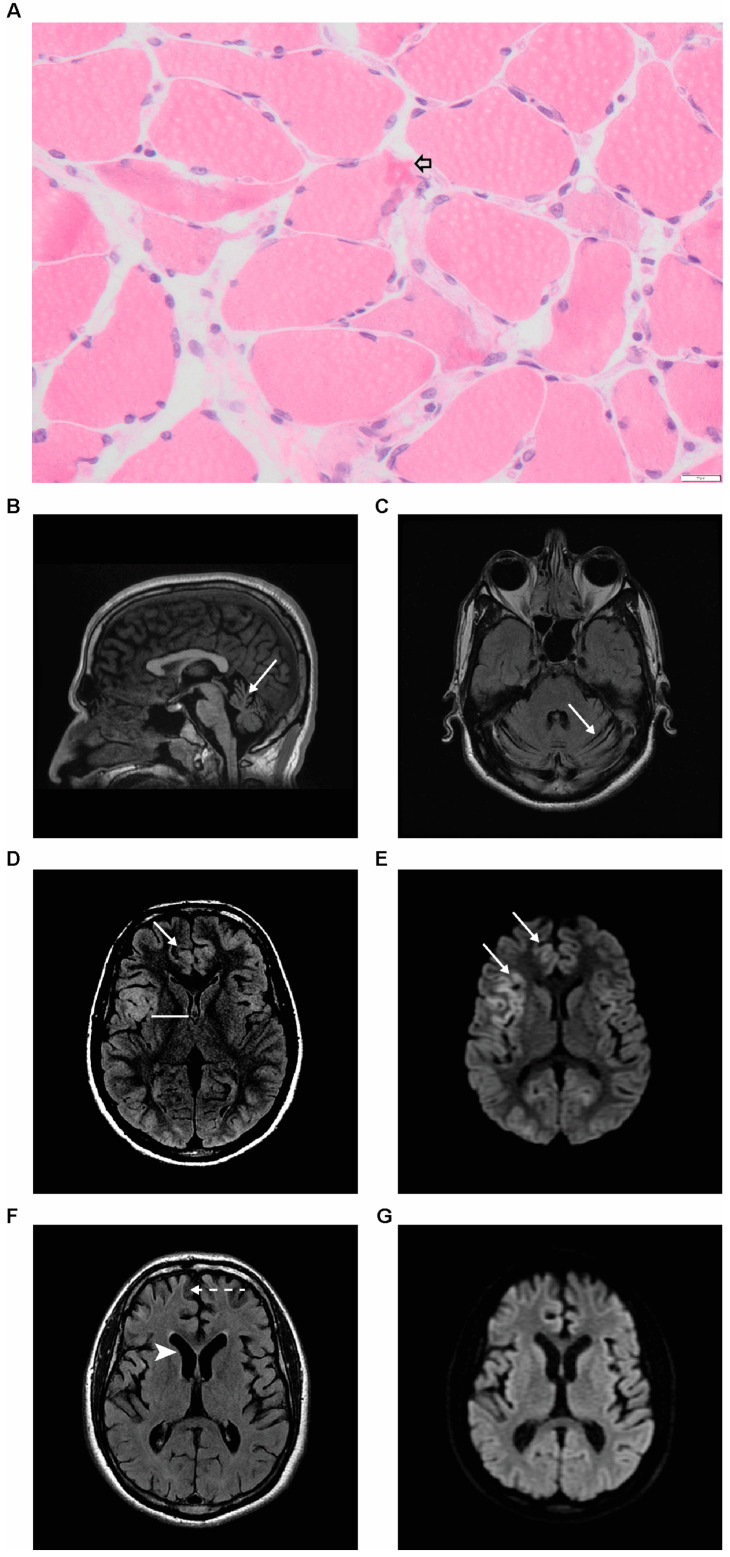
Muscle biopsy and neuroimaging findings in adult patient with NAXD deficiency. (**A**) Skeletal muscle showing rounded eosinophilic cytoplasmic bodies (arrow). Haematoxylin and eosin-stained section taken at 400× magnification (scale bar bottom right—20 μm). Initial MRI brain (first row) showing isolated moderate cerebellar volume loss (thin arrows in (**B**) and (**C**)). Second MRI (second row) performed 5 weeks later shows bilateral multifocal areas of cortical swelling and increased FLAIR signal intensity (block arrow in (**D**)). The corresponding areas demonstrate diffusion restriction (arrow in (**E**)), suggestive of cytotoxic oedema. Repeat MRI (last row) performed 3½ months after the second MRI, (**F**) near-complete resolution of cortical signal abnormality along with significant progressive global cerebral atrophy manifesting as cortical thinning (dash arrow in (**F**)) and ex-vacuo ventricular dilatation (also highlighted by the arrowhead in (**F**)), (**G**) demonstrates complete resolution of diffusion signal changes.

Serum and cerebral spinal fluid (CSF) testing for infective, inflammatory, autoimmune, paraneoplastic and metabolic causes including CSF 14-3-3 and RT-QuIC were unremarkable. CSF white blood cell count was <1 to 9 × 10^6^, CSF Tau was elevated at 1152 pg/mL, suggestive of a neurodegenerative process. Skeletal muscle from vastus lateralis showed the active degeneration and regeneration of fibres with some containing eosinophilic cytoplasmic bodies disrupting the myofibrillary matrix (Figure 1A). There was also type 2 atrophy, AMP deaminase deficiency and an occasional COX negative fibre. Densely centred filamentous cytoplasmic bodies and areas with disorganised myofibrils and normal appearing mitochondria were seen at electron microscopy. These findings, together with negative mitochondrial genetic testing, did not support a primary mitochondrial disorder. Pan-computerised tomography (CT) imaging was unremarkable for malignancy.

Initial brain magnetic resonance imaging (MRI) showed relatively normal hemispheric cortical morphology (Figure 1B) but moderate cerebellar atrophy (Figure 1B,C), documented on imaging 10 years prior and was attributed to chronic phenytoin administration. A repeat MRI scan 5 weeks later whilst intubated demonstrated multiple bilateral foci of cortical diffusion restriction and increased FLAIR signal intensity (Figure 1D,E), suggestive of cytotoxic oedema.

Electroencephalograms demonstrated severe encephalopathy and intermittent generalised or frontal epileptiform discharges (albeit often difficult to distinguish from frequent EMG artefact). Pulsed methylprednisolone and intravenous immunoglobulin (2 g/kg) for possible neuro-inflammatory causes did not lead to clinical improvement. Eventually, phenobarbitone burst suppression was induced following the failure of multiple anticonvulsants including midazolam and propofol to control frequent myoclonus and focal seizures. Epileptiform discharges recurred with weaning.

Following preliminary genetic results identifying a homozygous variant in NAXD (see below), high dose vitamin B3 (500 mg/day; 250 mg/day post-intensive care unit (ICU)), co-enzyme Q10 (150 mg/day), biotin (350 mg twice/day), L-arginine (750 mg three times/day) and L-carnitine (750 mg four times/day) were commenced based on a previous NAXD case report [4]. Although there was resolution of oral ulceration, encephalopathy and very frequent myoclonus remained unchanged. Periods of fever from intercurrent infections or possible drug reactions contributed to metabolic stress during this extended illness, particularly in intensive care. MRI imaging 3½ months later showed the near-complete resolution of cortical signal intensity, now with moderate global cerebral atrophy (Figure 1F,G). Findings were felt reflective of improvement in an inflammatory or metabolic process, or less likely, cortical epileptiform activity.

He lived for months with a tracheostomy, nasogastric tube feeding, anticonvulsant treatment and supportive care. His severe encephalopathic state remained unchanged and a decision was made for palliation.

### 2.2. Bioinformatics, Next-Generation Sequencing (NGS) and RNAseq Analysis

**Figure 2:**
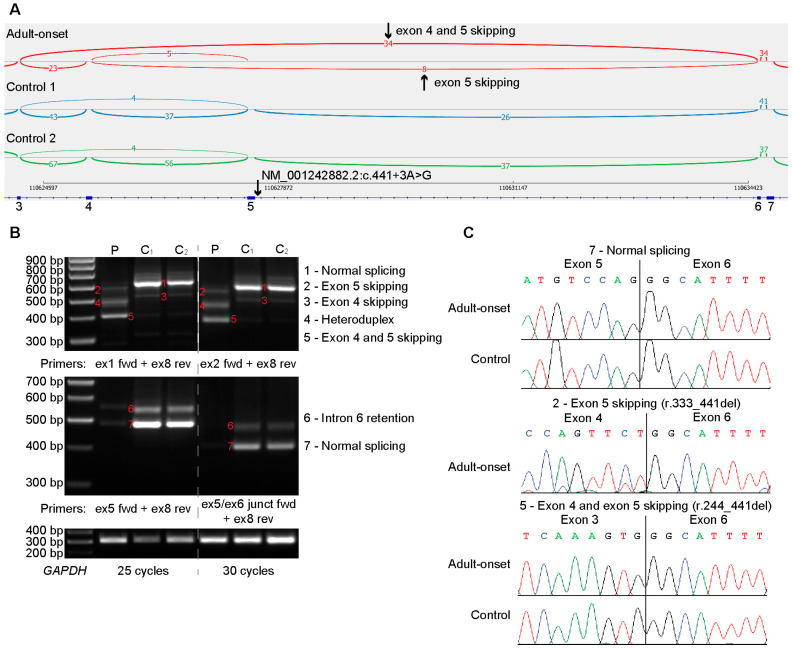
Transcriptomic and RNA splicing analyses of the *NAXD* c.441+3A>G variant. (**A**) Sashimi plots of RNA-Sequencing showing splicing of *NAXD* exons 3–7 in blood RNA from the adult patient (red) and two controls (blue, green). Exon 5 and exon 4–5 skipping is only detected in the adult sample and absent from controls. No annotated exon 5–6 splicing is detected in the adult sample. Minimum splice junction count = 3; control 1 = male, 34 years; control 2 = male, 8 years. (**B**) Confirmatory RT-PCR detects exon 4 and exon 4–5 skipping in the adult sample using a forward (fwd) primer in exon 1 or exon 2 and a reverse (rev) primer in exon 8 (ex1 fwd + ex8 rev; ex2 fwd + ex8 rev). Trace levels of canonical exon 5–6 splicing are detected in the adult samples using a forward primer in exon 5 or to the exon 5–exon 6 splice junction and a reverse primer in exon 8 (ex5 fwd + ex8 rev; ex5/ex6 junction fwd + ex8 rev). Amplification of GAPDH demonstrates cDNA loading. Lanes: Adult patient (P), Control 1 (C1) (male, 35 years), Control 2 (C2) (male, 36 years). (**C**) Sanger sequencing chromatograms of gel-excised RT-PCR amplicons showing exon 4 and exon 4–5 skipping, and canonical exon 5–6 splicing.

Rapid whole genome sequencing identified a homozygous variant in *NAXD* [Chr13(GRCh37):g.111279897A>G; NM_001242882.1:c.441+3A>G:p.?], absent in ClinVar and Genome Aggregation Databases. In silico tools concordantly predict that c.441+3A>G weakens or ablates the donor splice site of NAXD intron 5 (Supplementary Figure S1). Studies of *NAXD* pre-mRNA splicing using blood RNA show a near loss of NAXD transcripts with canonical exon 4-5-6 splicing, with no reads detected by RNA-Seq (Figure 2A) and only a faint band using reverse transcription polymerase chain reaction (RT-PCR) (Figure 2B, note faint band 7). c.441+3A>G causes the skipping of exons 4 and 5 (r.244_441del;p.Ala83_Gly148del), removing 66 conserved amino acids from the carbohydrate kinase domain, or the skipping of exon 5 (r.333_441del; p.Asp112Alafs*67), inducing a frameshift encoding 66 amino acids and a premature stop codon resulting in nonsense-mediated decay.

The trace levels of correctly spliced *NAXD* transcripts, reproducibly detected with several PCR primer pairs (Figure 2B), support a severe deficiency, but not complete absence, of NAXD. *NAXD* generates mitochondrial and cytosolic isoforms, with distinct clinical phenotypes dependent on whether the pathogenic variant exclusively affects the mitochondrial *NAXD* isoform, or both isoforms, termed ‘combined NAXD’ cases [1]. The mis-splicing events affect exon 4 and exon 5 (Figure 2C). While exon 4 is alternatively spliced, exon 5 is a canonical exon present in both cytosolic and mitochondrial NAXD transcripts expressed in the brain and blood (Supplementary Figure S2). The phenotype of the affected individual is consistent with ‘combined NAXD’ paediatric cases who also presented with neurological defects, seizures and skin lesions [1].

### 2.3. Fibroblast Proteomic Analyses in NAXD Patients

Fibroblast oxidative phosphorylation (OXPHOS)complex-I and complex-IV dipstick analyses were unremarkable in the proband, in contrast to two previous paediatric cases, perhaps reflecting limitations in assay sensitivity [2]. The lack of a reliable NAXD antibody prevented protein level analysis by Western blot [2]. Proteomics is more sensitive than respiratory chain enzymology in detecting mitochondrial defects in patients with mitochondrial diseases [5]. We conducted quantitative proteomic analysis of the fibroblasts from our adult patient, control subjects and four previously reported paediatric NAXD patients [2]. No NAXD-specific peptides were detected in this individual’s fibroblasts (Supplementary Figure S3), consistent with the near-deficiency of NAXD indicated by the RNA studies. Importantly, NAXD peptides were readily identified in the control fibroblasts, suggesting the absence of detectable NAXD in the adult proband is not due to the expression of NAXD at the lower limit of detection or other instrumentation-related problems (Supplementary Figure S3). A reduction in NAXD peptides in two paediatric cases [2] supported predictions based on the pathogenic variant (Table 1). These results are strongly suggestive of a near absence of NAXD protein in our adult patient.

**Figure 3:**
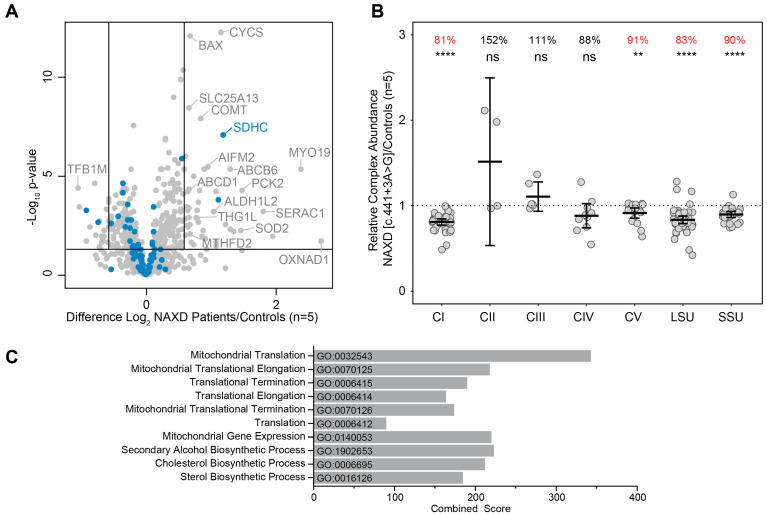
Fibroblast proteomic analysis of the proband with the *NAXD* c.441+3A>G variant. (**A**) Volcano plot showing protein abundances of mitochondrial proteins (based on MitoCarta3.0 annotations) detected through quantitative proteomics in the fibroblasts from all five NAXD cases relative to healthy controls (n = 5). Horizontal line represents *p* = 0.05 and the vertical lines represent fold changes of ± 1.5. Blue = OXPHOS subunits. (**B**) Relative complex abundance (RCA) profiles from quantitative proteomics revealed a significant reduction in mitochondrial OXPHOS complex subunits (CI and CV) and mitoribosomal proteins (large subunit ‘LSU’ and small subunit ‘SSU’) in fibroblasts from the adult onset NAXD case against controls (n = 5). Individual protein subunits are represented by a single dot. Overall mean values of subunits within each complex, which have been normalised to mitochondrial content levels, are represented by the middle bar. Upper and lower bars represent 95% confidence intervals. The mean ratio of each complex is shown as a percentage value on the top of each group. ns; *p* > 0.05, **; *p* ≤ 0.01, ****; *p* ≤ 0.0001. (**C**) Gene ontology (GO) terms for top ten biological processes enriched in cellular proteins decreased in abundance (*p* < 0.05, fold change −1.5) within three or more NAXD cases analysed by quantitative proteomics. Biological processes were determined by Enrichr [7,8] and ranked by adjusted *p*-value. The combined score, determined by multiplying the *p*-value and z-scores, is shown on the X-axis (full list in Supplementary Table S2).

A side-by-side comparison of the fibroblast whole-cell proteomic profiles quantified a striking ‘mitochondrial distress signature’ shared by all 5 NAXD cases (Supplementary Figure S4). A significant number of dysregulated proteins (Figure 3A, Supplementary Table S1) were identified, including the elevation of four proteins related to mitochondrial apoptotic processes (SOD2, BAX, CYCS and AIMF2), reduction in complex-I, complex-IV and small and large mitoribosome subunits (Figure 3B, Table 1 and Supplementary Figure S5), consistent with reports of mitochondrial defects in NAXD deficiency cases [2,6]. Muscle biopsies demonstrated the deficiency of AMP deaminase, which is part of the purine nucleotide cycle. It would be interesting to investigate whether NADHX directly inhibits AMP deaminase, and proteomics may provide unique insight into cellular dysfunctions subsequent to NAXD deficiency.

Enrichment analysis of cellular proteins with decreased abundance across the majority of NAXD cases identified several mitochondrial biological processes, highlighting the important role of NAXD within the mitochondria. Other processes related to alcohol and lipid biosynthesis were also listed, indicating possible additional functions of NAXD (Figure 3C, Supplementary Table S2). An enrichment analysis of proteins with increased abundances did not clearly identify biological processes directly related to NAXD functioning (Supplementary Table S2).

### 2.4. Fibroblast Metabolic Analyses in NAXD Patients

**Figure 4:**
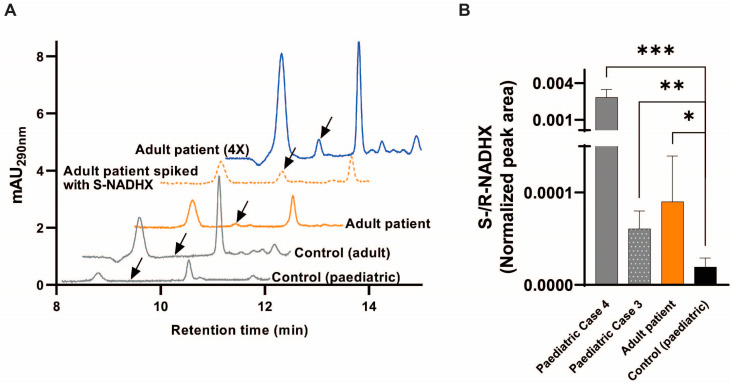
Fibroblast metabolic analysis of the proband with the *NAXD* c.441+3A>G variant. (**A**) HPLC-UV analysis of fibroblasts grown under standard cultivation conditions shows the presence of the damaged metabolite S-NADHX in the adult patient fibroblasts but not in adult or paediatric control lines. Chromatograms from a representative experiment are shown with the black arrows indicating the peak eluting at the retention time of standard S-NADHX. The orange dotted trace was obtained by analysis of the adult patient fibroblast extract spiked with 2 µM standard S-NADHX. The blue trace, indicated as adult patient (4X), was obtained by analysis of an adult patient fibroblast extract derived from an experiment in which cultures were launched by seeding four times more cells as in our standard protocol (1 × 10^6^ cells instead of 2.5 × 10^5^ cells). (**B**) ICMS analysis of fibroblasts cultured under galactose stress also showed increased S/R-NADHX levels in patient cells compared to control cells. Cyclic-NADHX forms were also detected at higher levels in patient fibroblasts (Supplementary Figure S6). Peak areas were normalised to internal standard (IS) areas. All values are means ± SD of four independent replicates. Statistical significance was calculated using an equal variance, unpaired Student’s *t*-test. * *p* < 0.05, ** *p* < 0.01, *** *p* < 0.001.

As NAXD deficiency leads to the accumulation of damaged forms of NADH [2], we assessed the presence of NADHX [9,10] in the fibroblasts from our adult proband using HPLC-UV (Figure 4A). Whereas NADHX was undetectable in the control fibroblasts in the standard medium, it was present in the adult NAXD fibroblasts. In the galactose medium, which was previously shown to hamper growth of NAXD fibroblasts [2], we also detected NADHX in our adult proband using mass spectrometry-based analyses, at levels that were significantly above the ones measured in the fibroblasts from a control subject and similar or below the ones measured in the paediatric NAXD patients (Cases 3 and 4 [2]; Figure 4B, Supplementary Figure S6). Accumulation of NADHX to different degrees in NAXD patients may be attributed to variable residual NAXD function dependent on the pathogenic variant/s, and potentially variable compensation mechanisms. Investigation of additional stress triggers (e.g., increased cultivation temperature) may further enhance the use of NADHX as a biomarker for NAXD dysfunction in fibroblasts.

## 3. Discussion

In conclusion, we showed that *NAXD* c.441+3A>G induces the mis-splicing of the majority of *NAXD* transcripts, leaving only trace levels of canonically spliced *NAXD* mRNA. Concordantly, quantitative proteomics showed that NAXD peptides in the fibroblasts from this adult were absent or below the limit of detection and LCMS analyses showed the accumulation of various forms of damaged NADH in the patient fibroblasts. We have uncovered shared mitochondrial proteomic signatures between the adult and paediatric NAXD cases, with reduced levels of complexes I and IV, the mitoribosome and upregulation of mitochondrial apoptotic pathways. We speculate that head trauma in adults, in addition to paediatric head trauma (Case 1 [2]), fever or illness in children, may precipitate neurometabolic crises associated with pathogenic *NAXD* variants, although one will be more confident of the former if the precipitant is also identified in future cases. The partial amelioration in the adult patient after B3 treatment, alongside preliminary niacin-based n-of-1 interventions for NAXD and NAXE deficiency cases (reviewed in [1,11]) provide evidence that early intervention may attenuate the severity of the injury or illness. Lastly, our work highlights the importance of considering rapid WGS for adults with likely monogenic disorders in ICU to aid in diagnostic decisions, with these benefits already demonstrated in a paediatric setting [12].

## 4. Materials and Methods

### 4.1. Ethics

All procedures followed were in accordance with the ethical standards and approved by the Human Research Ethics Committee of the Royal Children’s Hospital (HREC/67401/RCHM-2020 and HREC/16/MH251) and in accordance with the Helsinki Declaration of 1975, as revised in 2000. Written informed consent was obtained from the carer on behalf of the patient.

### 4.2. Cell Culture

Primary cultures of fibroblasts from Individual A1131048 and unrelated paediatric and adult control fibroblasts were established from skin biopsies as previously described [13]. Fibroblasts were cultured in basal medium (high-glucose DMEM (Gibco, Sydney, Australia) with 10% fetal bovine serum (Gibco, Sydney, Australia), 100 units/mL penicillin and 100 µg/mL streptomycin) at 37 °C with 5% CO_2_. All fibroblast control cell lines were established in-house and were established from paediatric individuals without any suspected genetic disorders.

### 4.3. Whole Genome Sequencing

DNA was extracted from blood, purified and its quality checked as previously reported [14]. Whole genome sequencing (WGS) was performed using massively parallel sequencing (NexteraTM DNA Flex Library Prep kit, NovaSeq Sequencers, Illumina, San Diego, CA, USA) with a mean target coverage of 30× and a minimum of 90% of bases sequenced to at least 10x for nuclear DNA (nDNA) and a minimum of 800× mean coverage for mitochondrial DNA (mtDNA). Data were processed, including read alignment to the reference genome (GRCh38) and the revised Cambridge Reference Sequence (rCRS) mitochondrial genome (NC_012920.1). Variant calling for nDNA was performed using Cpipe [15] or the functionally equivalent analysis with the Illumina Dragen System. For mtDNA, variants were called using Mutect2. For nDNA, variant analysis and interpretation within the selected target region (RefSeq genes ±1 kb) was performed using Agilent Alissa Interpret (Agilent, Santa Clara, CA, USA). Variants were annotated against all RefSeq gene transcripts and reported in accordance with HGVS nomenclature. Copy number variants (CNVs) were screened for using an internal CNV detection tool, CxGo [16]. For mtDNA, a custom in-house analysis pipeline was used to detect large deletions and annotate the VCF file with variant information. An mtDNA variant was considered as homoplasmic or apparently homoplasmic when it was present in at least 99% or 97%, respectively, of sequence reads aligned to the genomic position. Genes with proven disease association were considered during routine analysis. Curation of nDNA variants was phenotype-driven with pre-curated or custom gene lists used for variant prioritisation. Classification of nDNA and mtDNA variants was based on ACMG guidelines [17].

### 4.4. RNA Analysis

Whole blood was collected in a PAXgene (PreAnalytiX, Hombrechtikon, Switzerland) blood RNA tube and RNA was isolated using the PAXgene blood RNA kit according to kit instructions. All RNA Quality Scores were > 8.0 as determined by a LabChip GX Touch Nucleic Acid Analyzer (PerkinElmer Inc, Waltham, MA, USA). RT-PCR and Sanger sequencing performed as described previously with listed primers (Table 2) [18]. SuperScript IV (Invitrogen, Waltham, MA, USA) first-strand synthesis system was used to make complementary DNA (cDNA) from 500 ng of RNA according to kit instructions. Recombinant Taq DNA polymerase (Invitrogen, Waltham, MA, USA) and MasterAmp 2X PCR PreMix D (Epicentre Biotechnologies, Madison, WI, USA) were used for the PCRs. All PCR products were analysed on a 1.2% agarose gel. Bands were manually excised from an agarose gel with a scalpel and the cDNA was purified using the GeneJET gel extraction kit (Thermo Scientific, Waltham, MA, USA) according to the manufacturer’s instructions.

Replicate RNA sequencing libraries were prepared from 300 ng of RNA using Illumina Stranded Total RNA Prep with Ribo-Zero Plus (Illumina, San Diego, CA, USA). Sequencing was performed on the NovaSeq 6000 (300 cycles) (Illumina, San Diego, CA, USA), to produce at least 120M 150 bp paired-end reads per sample. Reads were aligned to the GRCh38 reference genome using STAR-2.7.8a [19] and visualised on Integrative Genomics Viewer [20].

### 4.5. Quantitative Mass Spectrometry and Data Analysis

Cellular pellets of fibroblasts from each of the five NAXD patient cell lines and five individual fibroblast control cell lines were collected. Each pellet was resuspended in 5% SDS and 50 mM triethylammonium bicarbonate and the concentration of total protein quantified for each sample using the Pierce BCA protein assay kit (Thermo Scientific, Scoresby, Australia). For NAXD patient cell lines, 25 µg of protein was aliquoted in triplicates, excluding the paediatric *NAXD* [*c*.308C>T] patient which was analysed in duplicates. Control cell lines were aliquoted in single 25µg protein amounts. Samples were processed with S-trap micro spin columns according to the manufacturer’s instructions (Profiti, Fairport, USA), where samples were reduced with 40 mM chloroacetamide (Sigma, Darmstadt, Germany) and alkylated with 10 mM tri(2-carboxyethyl) phosphine hydrochloride (TCEP; BondBreaker, Thermo Scientific, Scoresby, Australia). Isolated proteins were digested at a 1:10 trypsin to protein ratio at 37 °C overnight and eluted peptides were dried down using a CentriVap Benchtop Vacuum Concentrator (Labconco, Kansas City, MI, USA).

Peptides were reconstituted in 45 µL 2% acetonitrile, 0.1% trifluoroacetic acid buffer and 2 µL was injected to the system for analysis by liquid chromatography (LC)-tandem mass spectrometry (MS/MS). Samples were run on an Orbitrap Eclipse mass spectrometer (Thermo Scientific, Scoresby, Australia) connected to an Ultimate 3000 HPLC (Thermo Scientific, Scoresby, Australia) and NanoESI interface, and data were collected in a data-independent acquisition mode using a previously described method [21]. The system was equipped with an Acclaim Pepmap nano-trap column (Dinoex-C18, 100 Å, 75 µm × 2 cm) and an Acclaim Pepmap RSLC analytical column (Dinoex-C18, 100 Å, 75 µm × 50 cm) (Thermo Scientific, Scoresby, Australia). Raw files were processed using Spectronaut (v.16.2.220903.53000, Rubin) where files were searched against a data-dependent acquisition library containing 150,106 peptide precursors identified from deeply fractionated control fibroblast samples. The search was conducted using Default Spectronaut BGS Factory settings with changes made to exclude single hit proteins and allow for all identified peptides to be considered for quantification by selecting ‘Major Group Top N’ and ‘Minor Group Top N’ options. Proteins were searched for using the UniProt reviewed human canonical and isoform database (42,360 entries). Resulting data were imported into Perseus (v.1.6.15.0) [22] for statistical analysis, where known contaminants were filtered out and mitochondrial proteins were annotated using MitoCarta 3.0 [23].

For generation of relative complex abundance (RCA) plots, MS2 intensity levels of mitochondrial proteins were exported and analysed in RStudio using in-house scripts previously described [14]. Initially, the abundances of individual complex I–V subunits and mitoribosome proteins normalised to account for differences in total mitochondrial content between patient and controls. This was performed by dividing individual abundances by the sum of total mitochondrial proteins within individual replicates. The differences in proteins between patients and controls was then determined by calculating log10 of the normalised mean across replicates for each protein, and subtracting each protein mean of the control from the patient, which was then linearised and plotted as a ratio. A paired t-test was conducted to calculate the statistical significance of each complex between the control and patients. The mean and standard deviation were calculated from MS2 quantities, as well as the confidence intervals which were calculated from the t-statistic for each complex.

Within Perseus, raw MS2 quantity levels were log2 transformed and profile plots were generated using the built-in ‘profile plot’ function. To generate volcano plots, proteins in both patient and control groups were filtered to contain at least 2 valid values within each group and again filtered for mitochondrial proteins. The data were normalised to total mitochondrial content using the ‘Subtract row cluster’ function in Perseus where mitochondrial content is defined as a presence of the protein in MitoCarta3.0, and a two-sided t-test was conducted. For the volcano plots depicting the fold change in proteins in each NAXD patient against controls, the built-in ‘scatter-plot’ function was used with protein significance set at ±1.5 fold change (log2 ± 0.585) and *p*-value = 0.05 (−log10 = 1.301). The volcano plot depicting the changes between all NAXD patient cases against controls was generated in the same way, except all NAXD patients were included as replicates as part of the same group prior to the t-test being conducted to observe consistent changes across all patients relative to controls.

An enrichment analysis was conducted to identify the presence of pathways within cellular proteins be significantly increased or decreased in abundance (*p* < 0.05 and fold change ±1.5) within at least three of the five measured NAXD cases (adult and paediatric onset), using the Enrichr software [7,8]. Terms were ranked by adjusted *p*-value and a combined score calculated by the software was plotted.

### 4.6. Metabolic (NADHX) Analysis

For metabolite extraction experiments, fibroblasts were grown in a desired medium (with either glucose or galactose) for 72 h. Typically, 2.5 × 10^5^ cells were seeded per well into standard 6-well plates (Nunclon Delta Surface, Thermo Fisher Scientific, Thermo Fisher Scientific, Erembodegem, Belgium), except otherwise stated. For metabolite extractions under galactose stress, fibroblasts were seeded in basal medium (high-glucose DMEM (Gibco [Thermo Fisher Scientific], Erembodegem, Belgium) with 10% fetal bovine serum (Gibco [Thermo Fisher Scientific], Erembodegem, Belgium), 100 units/mL penicillin and 100 µg/mL streptomycin), allowed to grow for 24 h, after which the medium was changed to a medium containing galactose (DMEM without glucose/serine/glycine supplemented with 10% dialysed fetal bovine serum, 4 mM glutamine, 25 mM galactose, 100 units/mL penicillin and 100 µg/mL streptomycin), and the cells were then allowed to grow for another 72 h. Cells were gently washed either with pre-heated (37 °C) phosphate-buffered saline (for HPLC-UV measurements) or with pre-heated 100 mM HEPES (for ICMS measurements). Metabolites were then extracted with addition of 750 µL pre-cooled (−20 °C) extraction fluid (4:1 methanol/20 mM Tris buffer, pH 8 for HPLC-UV measurements or 4:1 methanol/water for ICMS measurements) into the wells and immediately scraping cells using a cell scraper. The collected extract was kept in an Eppendorf tube placed on ice. To recover a maximum of material, another 250 µL of extraction fluid was added to each well and mixed with the original extract. The pooled extract was then centrifuged at 21,000× *g* for 5 min at 4 °C. The supernatant (625 µL) was added to a 2 mL Eppendorf tube prefilled with 500 µL chloroform (4 °C), followed by addition of 325 µL of either 20 mM Tris buffer, pH 8 (4 °C) for HPLC-UV measurements or water for ICMS measurements. After a vigorous vortex (for 30 s to 1 min), the suspension was centrifuged at 21,000× *g* for 5 min at 4 °C. The upper polar phase was then filtered through a PHENEX-RC (Phenomenex, Utrecht, The Netherlands) 4 mm syringe filter and lyophilised overnight. The samples were either used directly for metabolite analysis or stored at −80 °C until analysis.

The HPLC-UV method for S-NADHX measurement was adapted from established methods [8]. In short, a Shimadzu Nexera UHPLC system (Shimadzu, Kyoto, Japan) equipped with a photo-diode array and fluorescence detectors was used for the measurements. The samples were analysed immediately after their preparation one by one. The lyophilised extracts were resuspended in 15 µL of 10 mM Tris buffer (pH 8) and 10 µL of the sample was immediately injected onto a Polaris C18-A column (3.0 × 150 mm, 3 µm particle size, Agilent, Diegem, Belgium) connected to a SecurityGuard ULTRA C18 precolumn (for 3 mm ID columns, Phenomenex, Utrecht, The Netherlands). The mobile phase consisted of 50 mM ammonium acetate, pH 7.0 and compounds were eluted with acetonitrile (ACN) using the following gradient: 0 min, 0% ACN; 20 min, 5% ACN; 25 min, 7.5% ACN; 32 min, 90% ACN; 33 min, 0% ACN; 40min, 0% ACN (re-equilibration step). The trace obtained at 290 nm is presented. The NADHX standards were prepared in-house, as described previously [8], and used for compound identification in HPLC and ICMS (see below) analyses.

For the ICMS measurements, polar extracts were analysed as previously described [24] with the following adjustments. Dried polar metabolite pellets were reconstituted in 25 µL of nanopure water (Milli-Q Advantage A10; Merck, Overijse, Belgium) with an injection volume of 20 µL. For the ICMS system, we interfaced a Dionex ICS-6000+ ion chromatograph with a QExactive high resolution mass spectrometer (both Thermo Fisher Scientific, Erembodegem, Belgium). Guard, column, gradient settings and suppressor type remained unchanged [22]. The heated electrospray ionisation (HESI) settings were as follows: spray voltage of 2.500 kV, auxiliary gas temperature of 420 °C, seep gas flow rate of 0, auxiliary gas flow rate of 15 units and sheath gas flow rate of 50 units. Settings for the acquisition of MS1 data were: m/z scan range of 80–750, resolution of 17.5 K, automatic gain control (AGC) target of 1e6 and maximum injection time of 100 milliseconds. Data were acquired using two micro-scans. For the MS2 data acquisition, the settings were: resolution of 17.5 K, AGC target of 2e5, maximum injection time of 50 milliseconds, loop count 2, isolation window of 4.0 m/z and collision energy of 30 eV. Peak areas were integrated, normalised to the internal standard (adenosine-^13^C_10_,^15^N_5_ 5’-monophosphate) areas and exported to Microsoft Excel via the Thermo TraceFinder (version 5.1) software. Doubly charged negative ions (loss of two protons) were used for relative quantifications of cyclic-NADHX (forms 1 and 2) and S-/R-NADHX using m/z values of 331.55511 and 340.56039, respectively.

## Figures and Tables

**Table 1:** Summary of quantitative proteomic analysis in fibroblasts from adult-onset and paediatric NAXD cases.

Age of Onset	*NAXD* Variant (and In Silico Prediction)	NAXD Peptides Detected	Mitochondrial OXPHOS Respiratory Chain (% of Controls)					Mitoribosome (% of Controls)	
			CI	CII	CIII	CIV	CV	LSU	SSU
Paediatric Case 1 ^#^	c.839+1G>T: p(?) and c.922C>T: p.(Arg308Cys) (splicing and missense)	7 peptides	79 ****	164	91	97	91 *	62 ****	63 ****
Paediatric Case 2 ^#^	c.187G>A: p.(Gly63Ser) and c.948_949insTT: p.(Ala317Leufs*64) *(missense and stop loss)*	~1–2 peptides	81 ****	150	102	67 ****	91 *	82 ****	78 ****
Paediatric Case 3 ^#^	c.51_54delAGAA: p.(Ala20Phefs*9) (frameshift w/ NMD)	2 peptides	93 **	124	111	90	86 ***	85 ****	87 ****
Paediatric Case 4 ^#^	c.308C>T: p.(Pro103Leu) (missense)	9 peptides	85 ****	149	102	89	89 **	87 ****	89 ****
Adult (reported here)	c.441+3A>G: p.(?) (residual wild-type, premature truncation and in-frame deletion)	Not detected	81 ****	152	111	91	91 **	83 ****	90 ****
Control		9 peptides	100	100	100	100	100	100	100

Summary of quantitative proteomic analysis in fibroblasts from adult and paediatric NAXD cases. #; Previously reported cases [2]. CI, complex-I; CII, complex-II; CIII, complex-III; CIV, complex-IV; CV, complex-V; LSU, large subunit of mitoribosome; SSU, small subunit of mitoribosome; FC, fold-change; NMD, nonsense-mediated decay; *, *p* < 0.05; **, *p* < 0.01; ***, *p* < 0.001; ****, *p* < 0.00001.

**Table 2:** Primer sequences.

Primer	Sequence
Ex1-F	5ʹ-CAATCCGGGCTTGCAGAC-3ʹ
Ex1-F2	5ʹ-GTCGTTGTCGCATTGCTCTC-3ʹ
Ex2-F	5ʹ-GAAAGAGCGTTTTCGCTACG-3ʹ
Ex5-F	5ʹ-CCAATGCTGTTCATGAGGTG-3ʹ
In5-R	5ʹ-AGGCACACGGAACACTACAC-3ʹ
Ex5/6-F	5ʹ-CTTCTCAGAAATGTCCAGGGC-3ʹ
Ex7-R	5ʹ-CTGAACTCCACGTGGTTGG-3ʹ
Ex8-R	5ʹ-GCTGTCATCGCTGTCCATAG-3ʹ

## Data Availability

The ES, GS and full proteomics datasets have not been deposited in a public database because of privacy and ethical limitations but may be made available upon reasonable request.

## Supplementary Materials

The following supporting information can be downloaded at: https://www.mdpi.com/article/10.3390/ijms24043582/s1.
