# Supplementary material for: Severe NAD(P)HX Dehydratase (NAXD) Neurometabolic Syndrome May Present in Adulthood after Mild Head Trauma
Source: Int J Mol Sci. 2023 Feb 10;24(4):3582. doi: 10.3390/ijms24043582 (PMC9963268; doi:10.3390/ijms24043582)
Supplement: Supplementary file 1 [file ijms-24-03582-s001.zip › ijms-2145312-Figures S1-S6.pdf]

Supplemental Figures and Tables

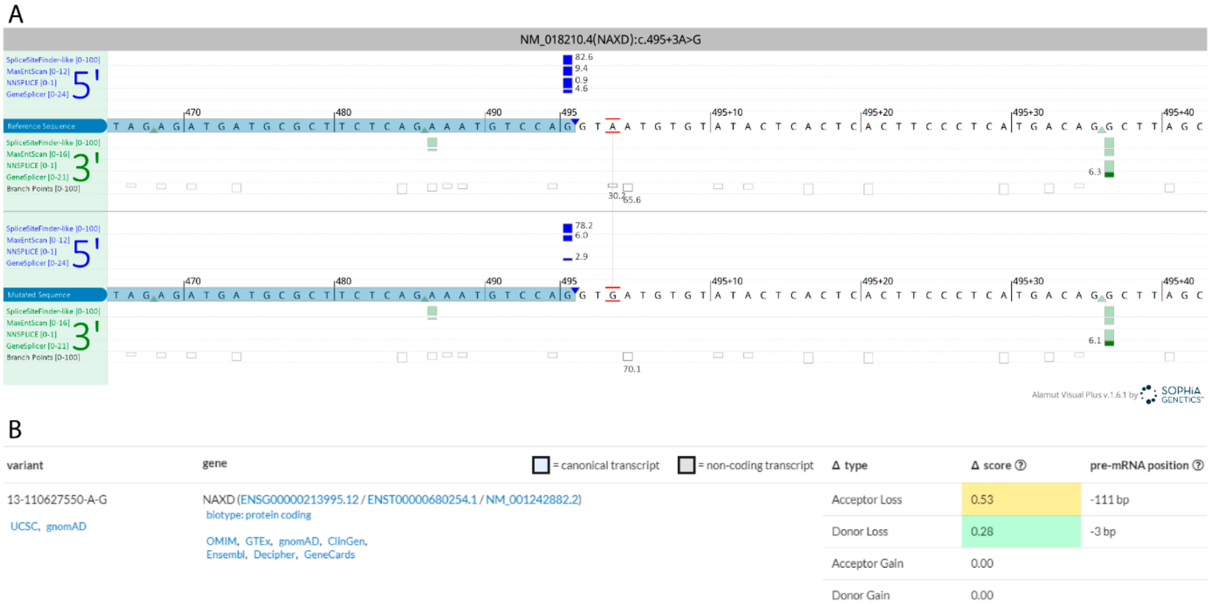

Supplemental Figure S1: Alamut Visual® and SpliceAI splicing predictions for the c.441+3A>G variant.

(A) NNSPLICE predicts the c.441+3A>G variant ablates the donor splice site of NAXD intron 5. SSF and MaxEntScan predict a decrease in splice site score (SSF 82.6 → 78.2 and MaxEntScan 9.4→ 6.0). GeneSplicer did not recognise the authentic donor to offer a prediction. **(B)** SpliceAI predicts a loss of Δ0.53 and Δ0.28 for the intron 4 acceptor and intron 5 donor (NM_001242882) respectively.

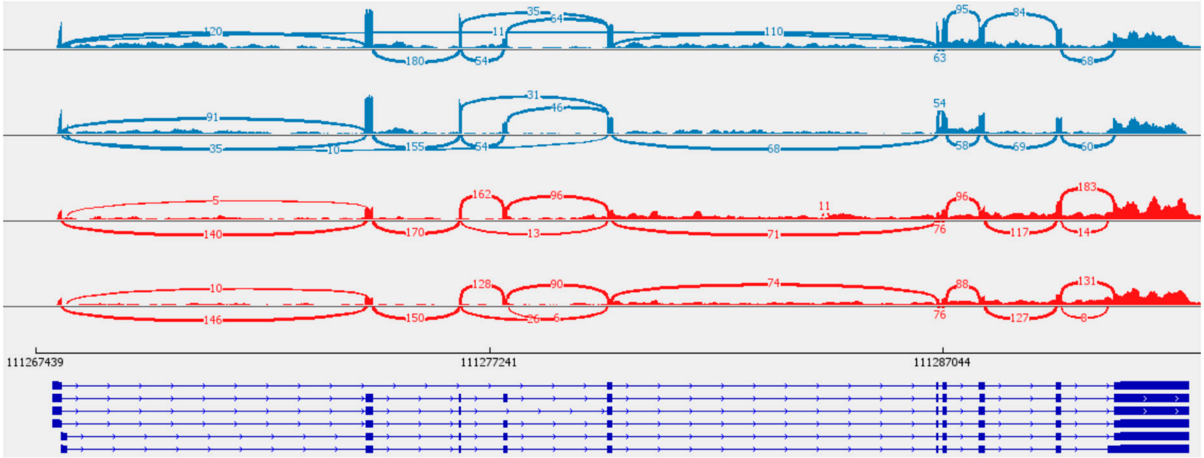

Supplemental Figure S2: RNA-seq coverage for *NAXD* in brain and blood.

Sashimi plot showing RNA-seq coverage for *NAXD* in mRNA from two brain samples (blue) and two blood samples (red). Exons 5 is a canonical exon in the predominant *NAXD* isoforms expressed in brain and blood. Natural exon 4 skipping is observed in *NAXD* transcripts expressed in brain and blood. RNA-seq data obtained from ENCODE and in-house data.

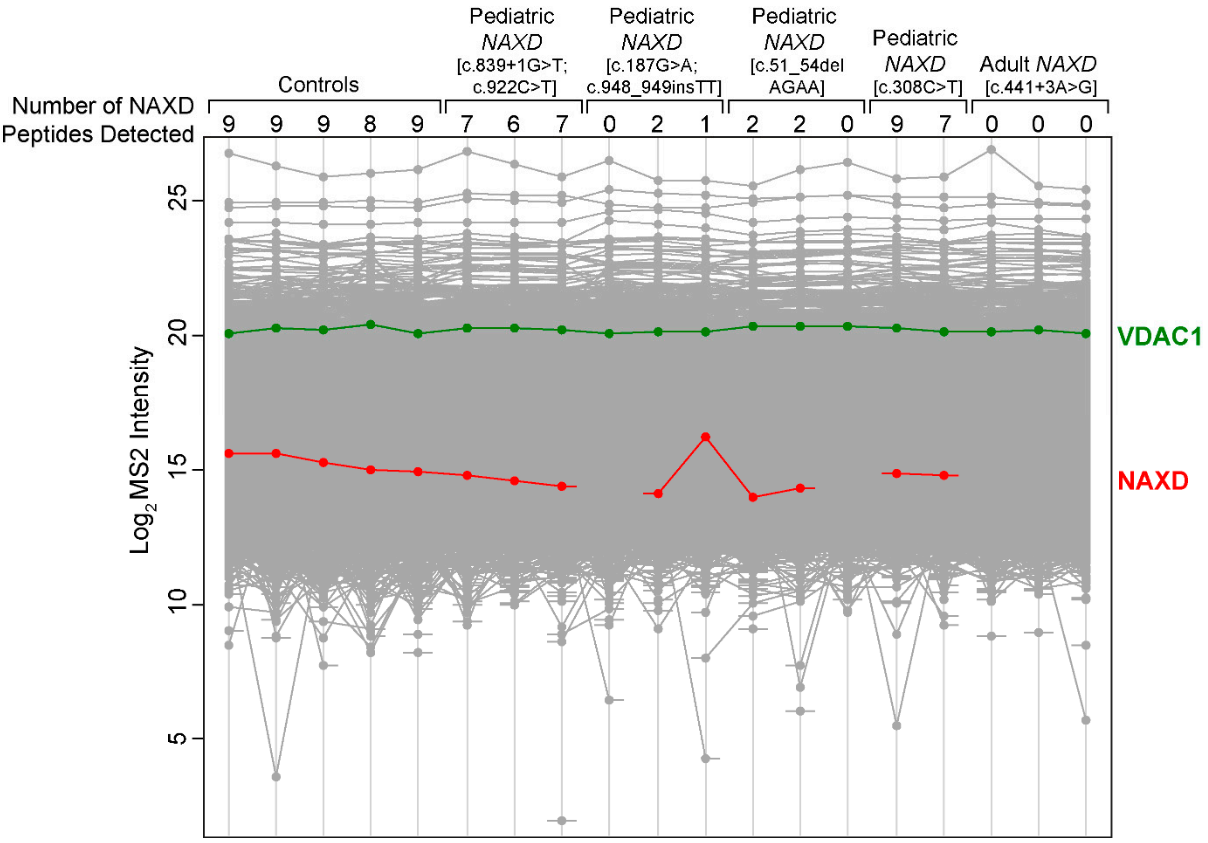

Supplemental Figure S3: MS2 quantities for NAXD across all quantitative proteomic samples.

Raw MS2 intensity levels of identified proteins from quantitative proteomic data analysed in the same sample batch were log2 transformed. Relative levels of NAXD protein within each sample were highlighted in red, with the number of unique NAXD peptides identified in each individual sample recorded at top of the graph. A mitochondrial marker protein VDAC1 was highlighted in green.

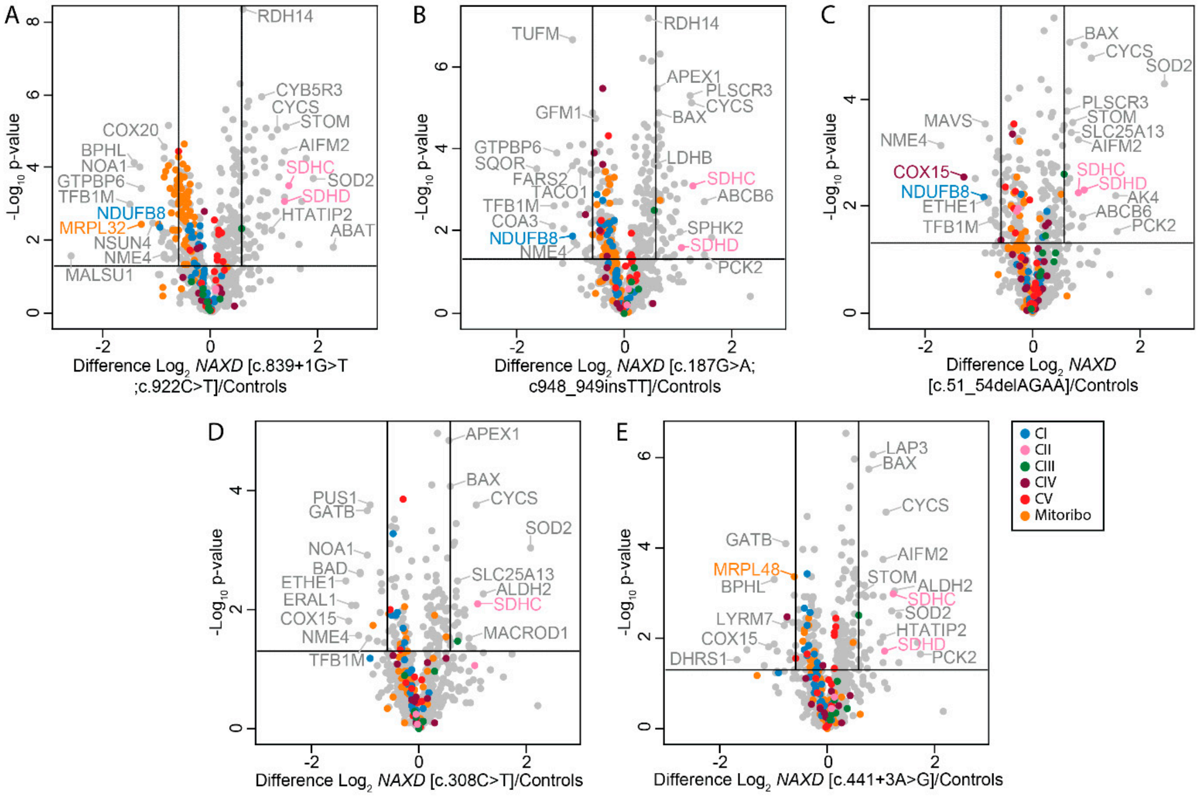

Supplemental Figure S4: Volcano plot of proteomics data in NAXD patients vs controls

Volcano plot showing protein abundances of mitochondrial proteins (based on MitoCarta3.0 annotation) detected through quantitative proteomics in NAXD patient fibroblasts against healthy controls (n=5) for previously reported paediatric cases **(A)** [c.839+1G>T;c.922C>T], **(B)** [c.187G>A;c.948_949insTT], **(C)** [c.51_54delAGAA], **(D)** [c.308C>T], and adult NAXD case **(E)** [c.441+3A>G]. Horizontal line represents p=0.05 and the vertical lines represent fold changes of ± 1.5. Blue = Complex I subunits. Pink = Complex II subunits. Green = Complex III subunits. Purple = Complex IV subunits. Red = Complex V subunits. Orange = Mitoribosome proteins

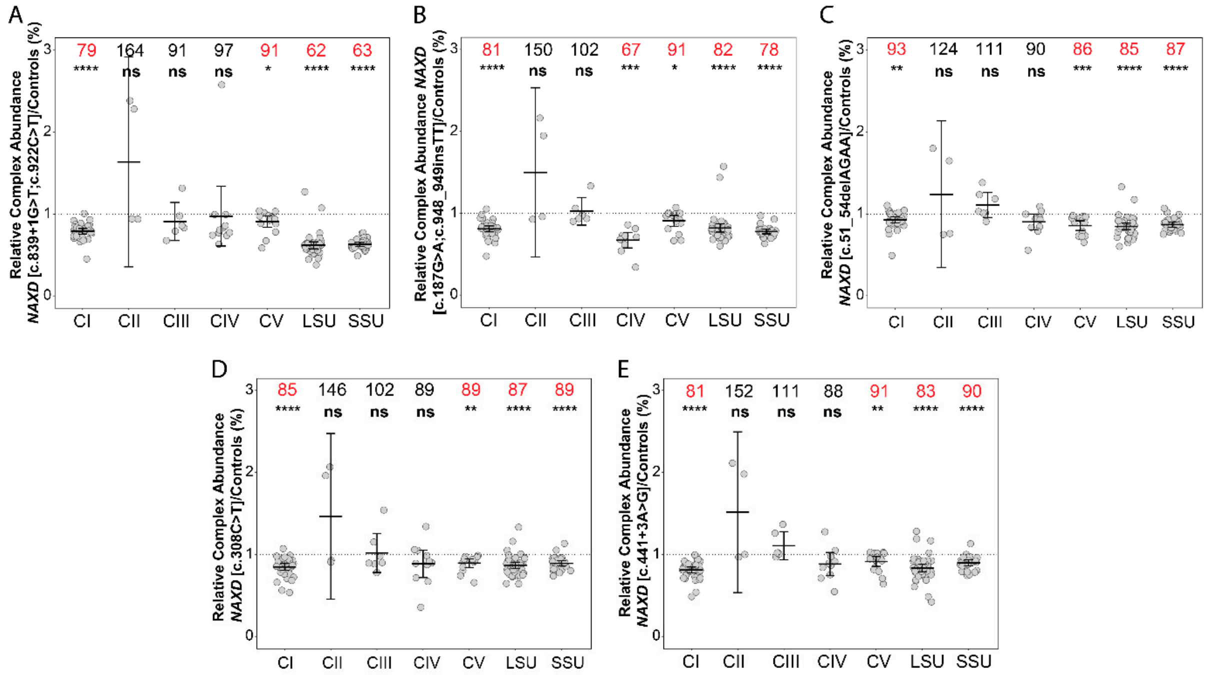

Supplemental Figure S5: Reduced mitochondrial OXPHOS proteins in NAXD cases.

Relative Complex Abundance (RCA) profiles from quantitative proteomics data depicting the ratio of OXPHOS complex subunits (CI-V) and Mitoribosome proteins (Large Subunit 'LSU' and Small Subunit 'SSU') in NAXD patients against controls (n=5). **(A)** Paediatric *NAXD* [c.839+1G>T;c.922C>T] patient, **(B)** Paediatric *NAXD* [c.187G>A;c.948_949insTT] patient, **(C)** Paediatric *NAXD* [c.51_54delAGAA] patient, **(D)** Paediatric *NAXD* [c.308C>T] patient, **(E)** Adult *NAXD* [c.441+3A>G] patient. Individual protein abundances were normalized to total mitochondrial content prior to ratios being calculated. Each dot represents a single protein subunit. The mean value of the subunits of each complex is represented by the middle bar, with upper and lower bars representing 95% confidence intervals. The ratio mean of each complex is also shown as a percentage value on top of each group. ns; $p > 0.05$, *; $p \leq 0.05$, **; $p \leq 0.01$, ***; $p \leq 0.001$, ****; $p \leq 0.0001$.

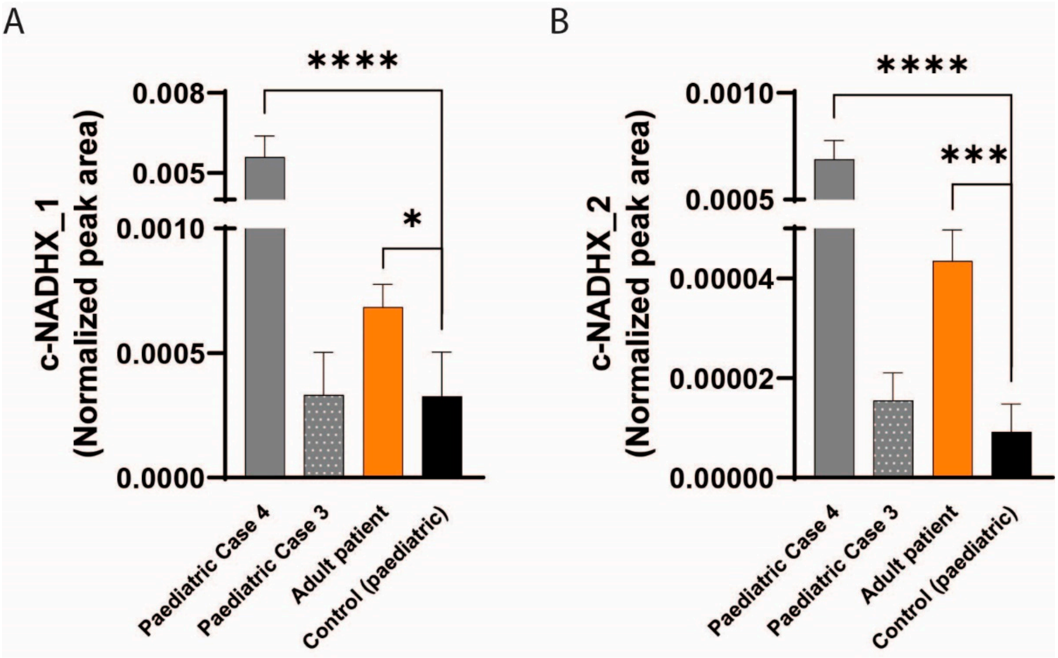

Supplemental Figure S6: NADHX metabolic analysis in adult patient under galactose stress growth conditions

Ion-chromatography coupled to mass spectrometry (ICMS) analysis of fibroblasts cultured under galactose stress also showed increased levels of cyclic-NADHX form 1 **(A)** and form 2 **(B)** in patient cells (Paediatric Case 4, Paediatric Case 3, Adult NAXD patient) compared to control cells. S/R-NADHX was also detected in patient fibroblasts (Fig. 4B). Peak areas were normalized to internal standard (IS) areas. All values are means ± SD of four independent replicates. Statistical significance was calculated using an equal variance, unpaired Student's t-test. *$p < 0.05$, ***$p < 0.001$, ****$p < 0.0001$.

Supplemental Table S1: Quantitative proteomic data for NAXD patient and control fibroblasts

Proteomics data on NAXD patients, including data used to generate Volcano plots and profile plots of log2-transformed intensities for cellular proteins in NAXD patient and control fibroblasts.

(See Excel file)

Supplemental Table S2: Full list of GO biological pathway enrichment data for proteins with significantly decreased and increased abundances measured within NAXD patients.

(See Excel file)
